# Near Miss or Standard of Care? *DPYD* Screening for Cancer Patients Receiving Fluorouracil

**DOI:** 10.3390/curroncol28010012

**Published:** 2020-12-18

**Authors:** Lauren E. Winquist, Michael Sanatani, Richard B. Kim, Eric Winquist

**Affiliations:** 1 London Health Sciences Centre and Schulich School of Medicine & Dentistry, Western University, London, ON N6A 5W9, Canada; lauren.winquist@lhsc.on.ca (L.E.W.); michael.sanatani@lhsc.on.ca (M.S.); Richard.Kim@lhsc.on.ca (R.B.K.); 2 Division of Medical Oncology, Department of Oncology, Western University, London, ON N6A 5W9, Canada; 3 Division of Clinical Pharmacology, Department of Medicine, Western University, London, ON N6A 5W9, Canada

**Keywords:** fluoropyrimidines, dihydropyrimidine dehydrogenase, cancer, adverse effects, single nucleotide polymorphisms, drug therapy

## Abstract

5-fluorouracil (5-FU) and its pro-drug capecitabine are widely used anticancer agents. Most 5-FU catabolism is dependent on dihydropyrimidine dehydrogenase (DPD) encoded by the *DPYD* gene, and *DPYD* variants that reduce DPD function increase 5-FU toxicity. Most DPD deficient patients are heterozygous and can be treated with reduced 5-FU dosing. We describe a patient with a genotype associated with near complete absence of DPD function, and severe and likely fatal toxicity with 5-FU treatment. The patient was treated effectively with alternative systemic therapy. Routine pretreatment *DPYD* genotyping is recommended by the European Medicines Agency, and guidelines for use of 5-FU in DPD deficient patients are available. However, outside the province of Quebec, routine pretreatment screening for DPD deficiency remains unavailable in Canada. It is likely our patient would have died from 5-FU toxicity under the current standard of care, but instead provides an example of the potential benefit of *DPYD* screening on patient outcomes.

## 1. Introduction

Fluoropyrimidines are chemotherapy agents used in the treatment of many solid tumors. This medication class includes 5-fluorouracil (5-FU) and its pro-drugs capecitabine and Tegafur that are converted into 5-FU in the body [1,2]. The adverse effects of fluoropyrimidines are numerous and well-described, ranging in severity from skin rash and mucositis to cardiac arrhythmia and bone marrow suppression. Notably, there is a 0.5–1% mortality rate associated with fluoropyrimidine treatment [1,2]. Fluoropyrimidine toxicity is thought to arise from the antimetabolic effects of the drug and its metabolites, which effectively but non-specifically affect replicating cells throughout the body. This allows for apoptosis of rapidly dividing cancerous cells, but also puts other tissues with rapid cell turn-over, such as mucous membranes and bone marrow, at risk.

The biochemical breakdown of 5-FU in the body is complex, but most of 5-FU catabolism is dependent on the enzyme dihydropyrimidine dehydrogenase (DPD). This enzyme is encoded by the *DPYD* gene, and the importance of *DPYD* genotypic variants in 5-FU metabolism and toxicity has been reviewed [2]. Both clinical and biochemical observations during the 1990s led to the association of hereditary DPD deficiency with severe fluoropyrimidine toxicity. The *DPYD* loss of function allele DPYD*2A was the first allele to be recognized and is present in 2% of Caucasian European populations. As genetic technologies improved, multiple alleles leading to decreased DPD function were characterized, and four clinically significant alleles are currently recognized: DPYD*2A (IVS14 + 1G > A, c.1905 + 1G > A, or rs3918290), DPYD*13 (c.1679T > G and c.2846A > T), and *DPYD* c.1129–5923C > G (Haplotype B3). Up to 8% of patients have reduced DPD activity and 0.1% completely lack DPD activity [2,3,4]. Heterozygosity for one reduced function *DPYD* allele accounts for the majority of partial DPD activity (Table 1). Most of such patients are considered intermediate metabolizers, are at higher risk for 5-FU toxicity, and reduced fluoropyrimidine dosing is recommended (Table 1) [5]. Patients with homozygous DPYD*2A or DPYD*13 (c.1679T > G) or compound heterozygotes of these loss of function alleles are considered poor metabolizers and are at high risk for severe and potentially fatal 5-FU toxicity [2]. If chemotherapy is necessary, an alternative non-fluoropyrimidine regimen is recommended. With their consent, we describe a patient with near complete DPD enzymatic deficiency treated with non-fluoropyrimidine-based therapy.

## 2. Case Presentation

A 53-year-old male underwent routine screening for colorectal cancer with a fecal immunochemical test. This test returned positive and he underwent further investigation including a colonoscopy, which identified a 4 cm non-obstructing ulcerated tumor in the rectum. Biopsy confirmed invasive adenocarcinoma. Routine bloodwork, serum carcinoembryonic antigen level, and whole body computed tomography and abdominopelvic magnetic resonance imaging were otherwise negative, and he was staged as cT3bN0 rectal adenocarcinoma. Neoadjuvant chemoradiation with concurrent oral capecitabine followed by low anterior resection was planned. Pretreatment *DPYD* genotyping was performed as part of a grant-funded institutional screening program. A whole blood sample was collected from the patient and DNA was extracted using the MagNA Pure Compact Instrument (Roche). DNA was assessed on a ViiA 7 real-time PCR system (ThermoFisher Scientific, Waltham, MA, USA) using TaqMan^®^ allelic discrimination assays (ThermoFisher Scientific) for *DPYD* c.1905 + 1G>A (assay ID: C__30633851_20), c.2846A>T (assay ID: C__27530948_10), c.1679T>G (assay ID: C__11985548_10), and c.1236G>A (assay ID: C__25596099_30). Positive controls were included in the genotyping assay. This testing revealed a compound heterozygous DPYD*2A (IVS14+1 G/A) and *DPYD* c.1236 G>A (Haplotype-B3) genotype identifying this patient as a “poor metabolizer” associated with severely decreased DPD enzymatic activity. In view of this, raltitrexed was administered concurrently instead of capecitabine with 50.4 gray external beam radiation given in 28 fractions. Raltitrexed is a direct thymidylate synthetase inhibitor not dependent on DPD metabolism [6]. This treatment was well tolerated, and following completion of chemoradiation, surgery with low anterior resection and loop ileostomy was performed. Surgical pathology showed a 2.0 cm poorly differentiated adenocarcinoma completely resected with clear surgical margins and evidence of partial tumor regression; 2 of 26 lymph nodes were involved with the metastatic tumor. The final pathological stage was ypT2N1b (stage IIIA). In view of the predicted near complete absence of DPD enzymatic deficiency, the usual recommendation for six months of postoperative adjuvant chemotherapy with 5-FU, folinic acid and oxaliplatin (FOLFOX) was modified to raltitrexed plus oxaliplatin. The patient has completed adjuvant chemotherapy without serious adverse effects, has had ileostomy reversal, and one year after starting treatment has no signs of cancer recurrence.

## 3. Discussion

In April 2020, the European Medicines Agency recommended that genotyping for *DPYD* loss of function alleles should be performed prior to the initiation of fluoropyrimidine treatment [3]. The results of these tests can then be used to aid clinical decisions, leading to fewer adverse events. Most patients with DPD deficiency are heterozygous for a reduced function allele. These patients are at higher risk for severe 5-FU toxicity but severity varies with the affected allele, and they can usually still receive 5-FU at a lower dose (Table 1). Limited data exist on the current use of *DPYD* testing in Canada, but it is our experience that routine *DPYD* testing prior to 5-FU treatment in Canada is not the standard of care at most Canadian centres other than our centre and the province of Quebec, where access to routine screening for DPYD*2A became available for clinicians in 2017 [7]. Many barriers exist in the implementation of such testing programs, including the lack of unified recommendations on the use of *DPYD* testing, the initial investment of resources to implement such a program, and the practical laboratory requirements for timely completion of the tests. However, our experience is that once such testing is in place, clinician uptake is rapid [8]. Although there is a cost to pre-emptive *DPYD* testing, routine *DPYD* genotyping has been reported to be cost effective in the Canadian setting [8].

*DPYD* variant alleles do not entirely explain the adverse effects of 5-FU, but they do identify a subset of patients at particularly high risk for severe or even lethal toxicity. Other methods to screen patients for reduced DPD activity have shown mixed results and include: measurement of pretreatment endogenous serum uracil concentrations; uracil/dihydrouracil-ratio; 5-FU degradation rate; and pharmacokinetically adjusted 5-FU dosing [9]. Other patient characteristics such as sex, age, body composition, and renal function also contribute to risk of severe 5FU toxicity and should be considered in dose-individualization strategies [9]. Uridine triacetate is an antidote for 5-FU overdose, and has been reported effective in DPD-associated 5-FU toxicity, and therefore could be used as a post-treatment mitigating strategy in DPD deficient patients [10]. However, it is not routinely available in most hospitals due to cost and, because optimal use is within 24 h of chemotherapy, it is unlikely most patients with DPD-associated toxicity would be recognized and treated soon enough to be used effectively.

## 4. Conclusions

Our case modestly demonstrates the potential benefits of a *DPYD* testing program. The incidence of *DPYD* poor metabolizers is low, but not negligible, approximately 1:1000 [3]. This genetic abnormality leads to complete or near complete DPD deficiency, and individuals with this deficiency are usually completely asymptomatic prior to treatment with a fluoropyrimidine. The use of fluoropyrimidine therapy in *DPYD* poor metabolizers causes severe 5-FU toxicity that can be fatal. The average clinician might encounter one such case in their entire career and be able to justify the fatal outcome simply to chance. Our case illustrates a patient for whom death from 5-FU toxicity under the current standard of care was a realistic outcome. Instead, this is a case that serves as a striking reminder of the potential impact that *DPYD* testing can have on patient outcomes.

## Figures and Tables

**Table 1:** Clinical recommendations for patients treated with fluoropyrimidines based on *DPYD* genetic testing [2].

Patient DRYF Genotype Scenarios	Effect on DPD Function and Clinical Implications	Intervention
2 *2A variants ^a^ OR 2 *13 variants OR 1 *2A variant and 1 *13 variant	No DPD activity Highest risk for severe, potentially fatal toxicity	Avoid use of fluoropyrimidines
1 *2A variant ^b^ OR 1 *13 variant PLUS 1 c2846A > T variant OR 1 c.1129-5923C > G variant	Severely decreased DPD activity Very high risk for severe, potentially fatal toxicity	Avoid use of fluoropyrimidines If no alternative treatments available, fluoropyrimidine dose should be strongly reduced by >75% of the starting dose with early measurement of drug levels to make sure the drug levels are not too high
1 *2A variant ^c^ OR 1 *13 variant OR 1 or 2 c2846A > T variants OR 1 or 2 c.1129-5923C > G variants OR 1 c.1129-5923C > G variant PLUS 1 c2846A > T variant	Decreased DPD activity Increased high risk for severe, potentially fatal toxicity	Reduce starting dose of fluoropyrimidines by 25–50%, followed by titration of dose based on toxicity observed in the first 2 cycles
None of the variants reported above are detected ^d^	Normal DPD activity Normal risk of toxicity	No indication to change dose or therapy Use dosage and administration per drug label

Note. Genotypes are reported. Table adapted from Amstutz et al. [5] Abbreviation: DPD, dihydropyrimidine dehydrogenase. ^a^ The highest risk of severe/life-threatening toxicity. ^b^ A high risk of severe/life-threatening toxicity. ^c^ An increased risk of severe/life-threatening toxicity. ^d^ A normal risk of severe/life-threatening toxicity.

## References

[1] Wigle T.J., Tsvetkova E.V., Welch S.A., Kim R.B. (2019). DPYD and fluorouracil-based chemotherapy: Mini review and case report. Pharmaceutics.

[2] Innocenti F., Mills S.C., Sanoff H., Ciccolini J., Lenz H.-J., Milano G. (2020). All you need to know about DPYD genetic testing for patients treated with fluorouracil and capecitabine: A practitioner-friendly guide. JCO Oncol. Pract..

[3] https://www.ema.europa.eu/en/medicines/human/referrals/fluorouracil-fluorouracil-related-substances-capecitabine-tegafur-flucytosine-containing-medicinal.

[4] Wörmann B., Bokemeyer C., Burmeister T., Köhne C.H., Schwab M., Arnold D., Blohmer J.U., Borner M., Brucker S., Cascorbi I. (2020). Dihydropyrimidine dehydrogenase testing prior to treatment with 5-fluorouracil, capecitabine, and Tegafur: A consensus paper. Oncol. Res. Treat..

[5] Amstutz U., Henricks L.M., Offer S.M., Barbarino J., Schellens J.H.M., Swen J.J., Klein T.E., McLeod H.L., Caudle K.E., Diasio R.B. (2018). Clinical Pharmacogenetics Implementation Consortium (CPIC) guideline for dihydropyrimidine dehydrogenase genotype and fluoropyrimidine dosing: 2017 update. Clin. Pharmacol. Ther..

[6] Barni S., Ghidini A., Coinu A., Borgonovo K., Petrelli F. (2014). A systematic review of raltitrexed-based first-line chemotherapy in advanced colorectal cancer. Anticancer Drugs.

[7] Jolivet C., Nassabein R., Soulières D., Weng X., Amireault C., Ayoub J.-P., Beauregard P., Blais N., Carrier C., Cloutier A.-S. (2020). Implementing DPYD*2A genotyping in clinical practice: The Quebec (Canada) experience. Oncologist.

[8] Wigle T.J., Povitz B., Teft W., Legan R., Lenehan J.G., Gulilat M., Nevison S., Kritzinger J., Punaganty V., Keller D. (2019). Prospective cohort study of the impact of hospital-wide dihydropyrimidine dehydrogenase (DPYD) genotype testing for fluoropyrimidine-based chemotherapy on adverse events and hospital costs. J. Clin. Oncol..

[9] Knikman J.E., Gelderblom H., Beijnen J.H., Cats A., Guchelaar H.-J., Henricks L.M. (2020). Individualized dosing of fluoropyrimidine-based chemotherapy to prevent severe fluoropyrimidine-related toxicity: What are the options?. Clin. Pharmacol. Ther..

[10] Ma W.W., Saif M.W., El-Rayes B.F., Fakih M.G., Cartwright T.H., Posey J.A., King T.R., von Borstel R.W., Bamat M.K. (2017). Emergency use of uridine triacetate for the prevention and treatment of life-threatening 5-fluorouracil and capecitabine toxicity. Cancer.

